# Cytokinesis in Suspension: A Distinctive Trait of Mesenchymal Stem Cells

**DOI:** 10.3390/cells14120932

**Published:** 2025-06-19

**Authors:** Bhavna Rani, Hong Qian, Staffan Johansson

**Affiliations:** 1 Department of Medical Biochemistry and Microbiology (IMBIM), Biomedical Center, Uppsala University, P.O. Box 582, 751 23 Uppsala, Sweden; 2 Department of Medicine Huddinge, Center for Hematology and Regenerative Medicine, Karolinska Institute, Karolinska University Hospital, 141 86 Stockholm, Sweden; hong.qian@ki.se

**Keywords:** MSC, cytokinesis, anchorage independence, ALIX, midbody

## Abstract

Mesenchymal stem cells (MSCs) have a broad clinical potential, but their selection and expansion on plastic cause unknown purity and phenotypic alterations, reducing therapy efficiency. Furthermore, their behavior in non-adherent conditions during systemic transplantation remains poorly understood. The sphere formation from single cells is commonly used to assess stemness, but MSCs lack this ability, raising questions about their anchorage dependence for proliferation. We investigated whether bone marrow-derived MSCs can complete cytokinesis in non-adherent environments. Primary *human* and *mouse* bone marrow-derived MSCs were synchronized in early mitosis using nocodazole and were cultured on soft, rigid, or non-adherent surfaces. Both *human* and *mouse* MSCs displayed an ALIX (abscission licensor) recruitment to the midbody 40–90 min post-nocodazole release, regardless of the substrate adherence. Cells maintained for 4hr in the suspension remained viable, and daughter cells rapidly migrated apart upon the re-adhesion to fibronectin-coated surfaces, demonstrating cytokinesis completion in suspension. These findings distinguish MSCs from fibroblasts (which require adhesion for division), provide a more general stemness feature, and suggest that adhesion-independent cytokinesis is a trait relevant to the post-transplantation survival and tissue homing. This property may offer strategies to expand MSCs with an improved purity and functionality and to enhance engraftment by leveraging cell cycle manipulation to promote an early extracellular matrix deposition at target sites.

## 1. Introduction

In multicellular organisms, anchorage dependence is a fundamental mechanism that regulates cell proliferation and ensures tissue organization [1]. Most mammalian cells require the attachment to the extracellular matrix (ECM) to proliferate and may undergo apoptosis or cell cycle arrest when detached or in contact with an unsuitable ECM [2,3]. This physiological defense mechanism prevents the proliferation of cells at inappropriate sites, thus preventing dysplastic growth. In contrast, certain normal stem cells, such as neuronal and mammary stem cells, can divide in a suspension and form spheroids from single cells [4]. Similar spheroid-forming cells have also been identified in tumors and are often referred to as cancer stem cells (CSCs) [5]. The ability of a single non-adherent cell to form a spheroid requires a resistance to anoikis (apoptosis due to lack of ECM), the ability to bypass the G1/S cell cycle checkpoint, and the capacity to complete cytokinesis without an integrin-mediated adhesion. Each of these properties depends on partly separate sets of signaling reactions downstream of the integrin-induced focal adhesion kinase (FAK) activation, i.e., the activation of AKT, CDK4/6-cyclinD, and PLK1, respectively [6]. The colony formation in suspension cultures can be enhanced by the presence of secreted or exogenously added extracellular adhesion proteins, such as fibronectin, collagen, and laminin [7,8], and a cell’s intrinsic ability to assemble its own extracellular matrix is thus an additional known factor affecting the colony formation capacity.

Anoikis and G1 arrest typically act as protective barriers against aberrant proliferation, thereby preventing the expansion of delocalized cells; however, a cytokinesis failure does not always halt proliferation. Cytokinesis is a tightly regulated and highly ordered process that ensures the proper physical separation of daughter cells and the maintenance of diploidy after mitosis. Failed cytokinesis can result in tetraploid cells (4N) that may continue dividing, leading to polyploidy and genomic instability—key contributors to tumorigenesis [9,10]. Cytokinesis begins during anaphase and progresses through several stages, including the cleavage furrow ingression, midbody formation, and eventually abscission by membrane fusion [11]. Errors at different stages of the multistep process give rise to various outcomes ranging from acute cell death to aneuploidy and cancer [12]. Thus, while cytokinesis is not a checkpoint, successful cytokinesis is essential for genomic integrity. In fibroblast and epithelial cell lines, integrin-induced signals are involved primarily in the final stage of cytokinesis, abscission, through a FAK/PLK1/Cep55 [13] pathway required for the maturation of the midbody, a large microtubule-rich protein complex located at the intercellular bridge that serves as a platform for recruiting proteins essential for the separation of the daughter cells [14,15]. Specifically, the FAK activity downstream of integrins by an unknown mechanism induces the binding of ALIX and TSG101 to CEP55 in the midbody, which in turn leads to the recruitment of the ESCRT III abscission machinery to the midbody [13,16].

However, several stem and stem-like cells appear to circumvent this anchorage dependency, raising important questions about the molecular mechanisms enabling such behavior. Understanding these mechanisms is crucial for both the therapeutic application of normal stem cells and the targeted treatment of malignant ones. Mesenchymal stem cells (MSCs) are of particular interest due to their great potential in regenerative medicine and as modulators of immune reactions. Unlike mammary stem cells, MSCs cannot form spheroids from non-adherent single cells, indicating that they are anchorage-dependent for one or more properties essential for proliferation [17]. Nevertheless, bone marrow MSCs can enter the circulation and migrate to distant damaged tissues to promote repair. However, the clinical efficacy of externally delivered MSCs is still limited by the low survival and engraftment of the cells [18]. A better understanding of how MSCs accomplish proliferation at targeted sites with a potential foreign environment could improve both their therapeutic utility and our ability to target similar mechanisms in cancer. In this study, we focused on investigating the cytokinesis ability of bone marrow-derived MSCs under non-adherent conditions. This aspect is critical for their proliferation and differentiation potential, as well as for ensuring their safe therapeutic use by maintaining normal cell ploidy.

## 2. Materials and Methods

### 2.1. Cell Culture

Primary *mouse* MSCs with a phenotype of CD45-TER119-CD44-/CD36+/SCA1+ were sorted by flow cytometry from bone marrow mononuclear cells (isolated by enzyme dissociation under ethical permit, as described previously) [19,20]. The cells were cultured in the Dulbecco’s modified Eagle Medium (1×) + GlutaMax1TM (DMEM, Gibco, Life Technologies, Bleiswijk, The Netherlands) supplemented with 10% fetal bovine serum (FBS, Gibco (A5256701), Life technologies, Grand Island, NY, USA), 100 U/mL penicillin, 0.1 mg/mL streptomycin, and 0.01 mM β-mercaptoethanol. Animal procedures were performed with approval from the local ethics committee (ethical number 15861-2018) at Karolinska Institute (Stockholm, Sweden). Primary *human* MSCs (NBM102) were isolated from bone marrow aspirates and cultured in StemXVivo^®^ MSC Expansion Media (CCM004, Biotechne, Minneapolis, MN, USA) supplemented with 100 U/mL penicillin and 0.1 mg/mL streptomycin. The cells were initially expanded in a hypoxic incubator with 1% CO_2_ and subsequently kept at 37 °C in a humidified atmosphere containing 5% CO_2_ for the experiments. The bone marrow aspirate was obtained from a healthy adult volunteer after informed consent was obtained. The sample collection was approved by the local ethical committee at Karolinska Institute, Stockholm (ethical permit 2012/1971-31/3).

### 2.2. Cell Synchronization

Cells were synchronized in early mitosis with nocodazole treatment (20 ng/mL) for 5 h, as detailed in the figure legends. Mitotic cells were then harvested using the shake-off method [21], in which loosely attached, rounded mitotic cells were detached by gently tapping the culture flasks. To release the cells from nocodazole-induced mitotic arrest, they were washed once with pre-warmed PBS (Gibco, pH 7.4), followed by two washes with the complete growth medium (total processing time approximately 20 min). The cells were subsequently seeded under adhesion-independent conditions using bacterial-grade Petri dishes coated with Pluronic F108 (10 mg/mL; NF Prill Poloxamer 338, D-BASF, Monheim, Germany) or under adhesion-dependent conditions using tissue culture plates or coverslips (18 mm) coated with fibronectin (40 μg/mL). For some experiments, mitotic mbm-MSCs isolated by the shake-off method were seeded on fibronectin covalently coupled to matrix plates with an elastic modulus of 64 kPa (stiff matrix plate) or 0.5 kPa (soft matrix plate) (SoftSubstrates™, MuWells, San Diego, CA, USA).

### 2.3. Live-Cell Imaging

Live-cell imaging was carried out using an inverted microscope (Nikon Eclipse Ti-U, Tokyo, Japan) equipped with a CCD camera (Andor multi-pixel sCMOS camera, Oxford Instruments, Belfast, Ireland) and a cell culture chamber maintained at 37 °C with a constant supply of humidified 5% CO_2_. The images were acquired using an automated motorized multi-position stage with 20× magnification objectives and a phase contrast filter of the time-lapse microscope in 8 or 10 min time intervals for the desired time periods. Videos were analyzed using NIS software version AR 4.30. (Nikon Eclipse Ti-U, Tokyo, Japan).

### 2.4. Re-Adhesion Assay

Mitotic cells isolated from either *mouse* bone marrow-derived mesenchymal stem cells (mbm-MSCs) or *human* bone marrow-derived mesenchymal stem cells (hbm-MSCs) were cultured for 4 h on Pluronic-coated dishes (10 mg/mL; F108 NF Prill Poloxamer 338, D-BASF, Germany) to allow cytokinesis to complete in suspension. After suspension incubation, the cells were re-seeded onto fibronectin-coated tissue culture dishes and subjected to live-cell imaging to monitor whether cytokinesis had been completed in suspension.

### 2.5. Immunofluorescence Staining

For the adhesive condition, mitotic cells were cultured on fibronectin-coated high precision coverslips of 18 mm (Cat # 0117580, no.1.5 H, Marienfeld, Lauda-Königshofen, Germany), while for the non-adhesive condition, cells were cultured on Pluronic-coated (10 mg/mL, F108 NF Prill Poloxamer 338, D-BASF, Germany) 10 cm bacterial plates (Sarstedt, Helsingborg, Sweden) and subsequently spun down onto glass slides using cytospin centrifugation (80× *g* for 1 min). Cells were then fixed with cold methanol at −20 °C for 20 min and washed twice with PBS for 5 min each. Following fixation, the cells were incubated in a blocking buffer containing 1% BSA (Fraction V; Roche Diagnostics, Mannheim, Germany) and 0.1% Tween-20 (P7949, Sigma Aldrich, St. Louis, MO, USA) in PBS. Slides were then incubated overnight at 4 °C with primary antibodies diluted 1:100 in the blocking buffer. The following primary antibodies were used: Aurora B (ab2254, ab3609; Abcam, Cambridge, UK) and ALIX (sc 53540, Santa Cruz Biotechnology, Inc., Dallas, TX, USA; purchased from AH diagnostics AB, Solna, Sweden). After primary antibody incubation, slides were washed thrice with 1× PBS and incubated for 1 h at room temperature with secondary antibodies Alexa Fluor 488-conjugated goat anti-rabbit and Alexa Fluor 594-conjugated goat anti-mouse (Invitrogen, Carlsbad, CA, USA) at 1:500 dilution in blocking buffer. After a final PBS wash, slides were mounted using a medium containing DAPI (4′,6-diamidino-2-phenylindole; Invitrogen). Both primary and secondary antibody incubations were performed in a humidified chamber.

Digital images were taken with a Nikon fluorescence microscope (Eclipse 90i, Nikon, Japan) outfitted with a DS-Qi1 monochromatic CCD camera. Immunostaining was examined for protein localization in the midbody, and quantification was carried out using FIJI (ImageJ) software version 2.16.0/1.54p (https://imagej.net/software/fiji/ (accessed on 21 May 2025)).

### 2.6. Quantification of Cytokinesis Completion and Failure

In the re-adhesion assay, isolated rounded mitotic cells were plated onto fibronectin-coated substrates, where they were subsequently flattened. Successful cell division during the prior suspension period was identified by the clear separation of daughter cells migrating away from each other as visualized by time-lapse microscopy. In case cytokinesis had failed, a single binucleated cell appeared instead. In fixed samples, the passing of the abscission licensing step was assessed by the presence of ALIX at the midbody.

### 2.7. Statistical Analysis

The statistical analyses were performed using Student’s *t*-test. *p*-values < 0.05 were considered significant. For all the experiments, 100–200 randomly selected cells per condition and for each time point were analyzed from each of the three independent experiments (n = 3). * *p* < 0.05, ** *p* < 0.01, *** *p* < 0.001, and **** *p* < 0.00001.

## 3. Results

### 3.1. Primary Mouse Bone Marrow MSCs Undergo Cytokinesis Independent of Matrix Stiffness

Integrin-mediated adhesion signaling can occur via two primary mechanisms: (1) the ligand-induced clustering of integrins and associated proteins [22,23] and (2) force-induced conformational changes in stretch-sensitive proteins within the adhesion complex (mechano-signaling) [24]. The murine mesenchymal stem-like cell line C3H 10T1/2 has been reported to complete cytokinesis independently of the integrin-induced mechano-signaling, successfully dividing on both stiff and compliant extracellular matrix (ECM) substrates [25].

**Figure 1:**
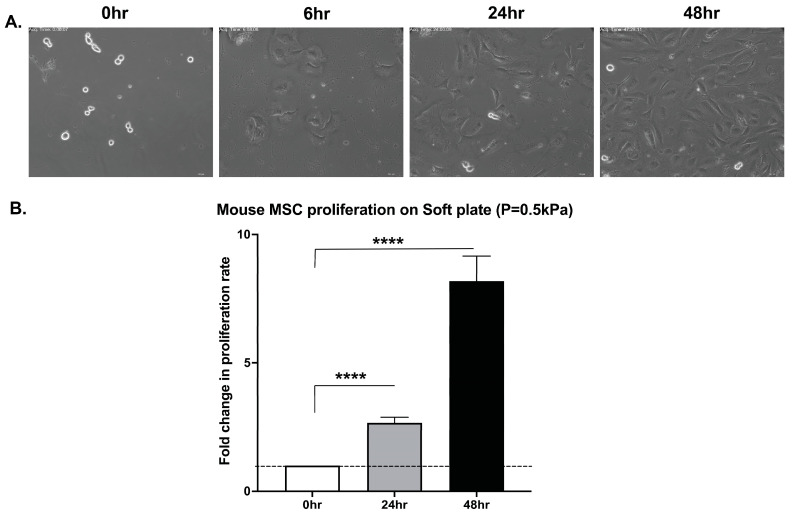
mbm-MSCs proliferate on compliant substrates. (**A**) Bright-field screen shots (from Video S1) of mitotic *mouse* MSCs cultured on compliant substrates (soft matrix plate 0.5 kPa), illustrating cell proliferation over time. Scale bar: 10 μm. (**B**) The accompanying bar graph depicts the fold change in the cell number at 0, 24, and 48 h relative to the initial seeding density (mean ± SEM, n = 3). The statistical significance was assessed by a paired two-tailed Student’s *t*-test; **** *p* < 0.00001.

**Figure 2:**
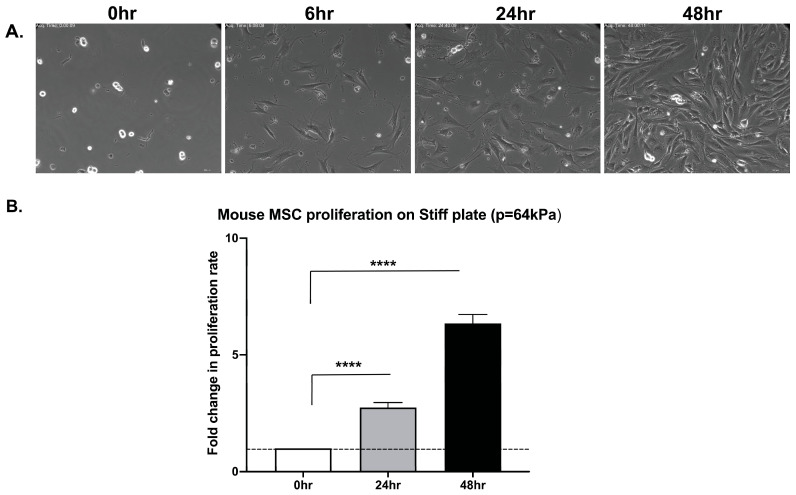
mbm-MSCs proliferate on rigid substrates. (**A**) Bright-field screen shots (from Video S2) of mitotic *mouse* MSCs cultured on stiff substrates (stiff matrix plate 64 kPa), illustrating cell proliferation over time. Scale bar: 10 μm. (**B**) Bar graph depicts the fold change in the cell number at 0, 24, and 48 h relative to the initial seeding density (mean ± SEM, n = 3). The statistical significance was assessed using a paired two-tailed Student’s *t*-test; **** *p* < 0.00001.

To determine whether this property is shared by primary *mouse* bone marrow-derived MSCs (mbm-MSCs, nonhematopoietic and non-endothelial cells sorted based on the phenotype of CD45-TER119-CD31-CD44-CD36+SCA1+), mitotic cells were sparsely seeded on fibronectin-coupled substrates with a defined stiffness: a compliant substrate (soft matrix plate 0.5 kPa) and a rigid substrate (stiff matrix plate 64 kPa). Live-cell time-lapse microscopy confirmed that mbm-MSCs were capable of completing mitosis and cytokinesis on both substrates (Figure 1 and Figure 2 and Videos S1 and S2). Notably, the cell morphology was different on the two substrates, and on the stiff matrix, “footprint” cell fragments were frequently torn off from the cells during their migration. The quantitative analysis of the proliferation over 48 h revealed comparable expansion rates on both the stiff and soft matrix, with approximately a 2.6-fold increase at 24 h and a 6.5- and 8.0-fold increase at 48 h, respectively, relative to the initial seeding density (Figure 1 and Figure 2). These findings indicate that primary mbm-MSCs can undergo cytokinesis and proliferate independently of mechanical signals derived from the integrin-mediated adhesion.

### 3.2. Midbody Maturation and Abscission Occur in Mbm-MSCs Without Adhesion to ECM

**Figure 3:**
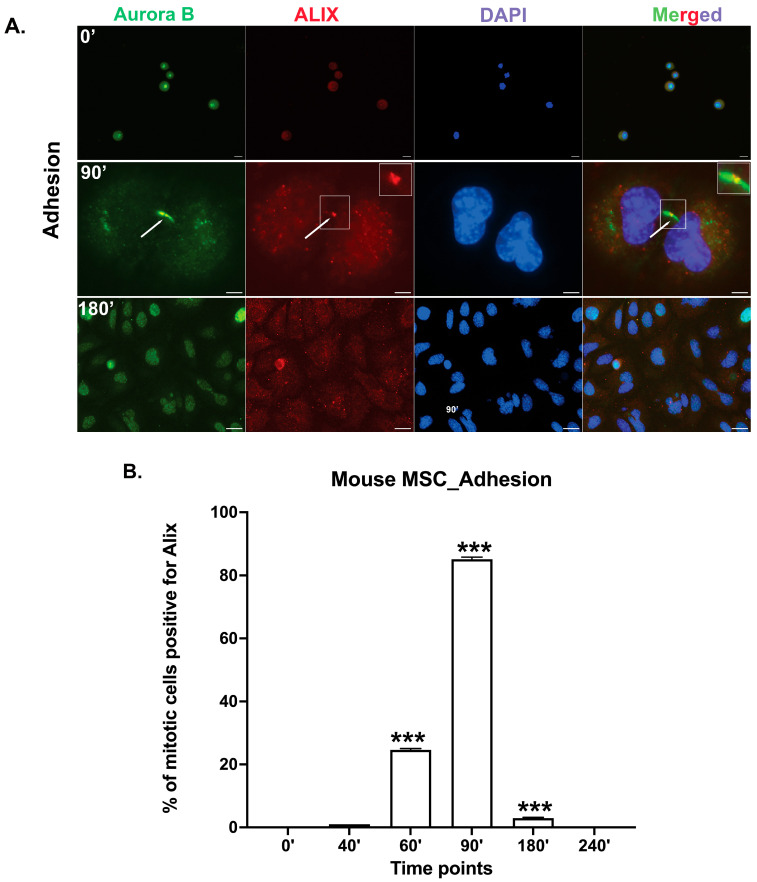
Cytokinesis progresses efficiently in mbm-MSCs under adherent conditions. (**A**) Representative immunofluorescence images of mbm-MSCs, showing the localization of the Aurora B kinase (green), an early midbody marker, and ALIX (red), an abscission-licensing factor, at indicated time points under adherent conditions. Nuclei were counterstained with DAPI (blue). White arrows indicate the midbody region during cytokinesis. The boxed area highlights a magnified view of the midbody region indicated by the arrows. Scale bar: 10 μm for 0 and 180 min and 2.5 μm for 90 min. (**B**) The quantification of the percentage of cells displaying the ALIX localization at the midbody over time. The fraction of cells that recruited ALIX to the Aurora B-positive midbody was measured over time under adherent conditions. ALIX was localized to the midbody in ~86% of cells at 90 min post-release from mitotic arrest, followed by a sharp decline to <2% by 180 min and a complete absence by 240 min. This temporal pattern is consistent with the progression through abscission and the successful completion of cytokinesis. Data represent mean ± SEM from n = 3 independent experiments. The statistical analysis was performed using an unpaired two-tailed *t*-test; *p* < 0.05 was considered statistically significant; *** *p* < 0.001.

Cytokinesis is the final stage of cell division, during which abscission is induced from the midbody. To evaluate whether primary *mouse* mbm-MSCs can complete cytokinesis independently of integrin-mediated adhesion signaling, cells were synchronized at the prometaphase using a nocodazole treatment. Mitotic cells were collected by mechanical shake-off and, after the nocodazole wash-out, replated onto either fibronectin-coated tissue culture dishes (adhesive condition) or Pluronic-coated bacterial-grade dishes (non-adhesive suspension condition). Cells were cultured and fixed at various time points (0, 40, 60, 90, 180, and 240 min) and immune-stained for Aurora B kinase, a marker of early midbody formation [26], and ALIX, a key regulator of the abscission phase of cytokinesis [13,14,16]. At the 0 min time point, Aurora B was localized to condensed chromatin, consistent with its role in early mitosis. As cells advanced through mitosis, Aurora B translocated to the midbody in both adherent and suspension conditions, demonstrating the progression into cytokinesis (Figure 3). Under adherent conditions, ALIX became detectable at the midbody in a small subset of cells by 40 min and was observed in approximately 86% of cells by 90 min, which is consistent with its recruitment during the abscission phase and similar to previous reports for fibroblasts [13]. By 180 min, the number of cells positive for Aurora B and ALIX at the midbody diminished, and by 240 min, midbody proteins were not detectable, indicating the completion of abscission and the removal of the midbody structure.

**Figure 4:**
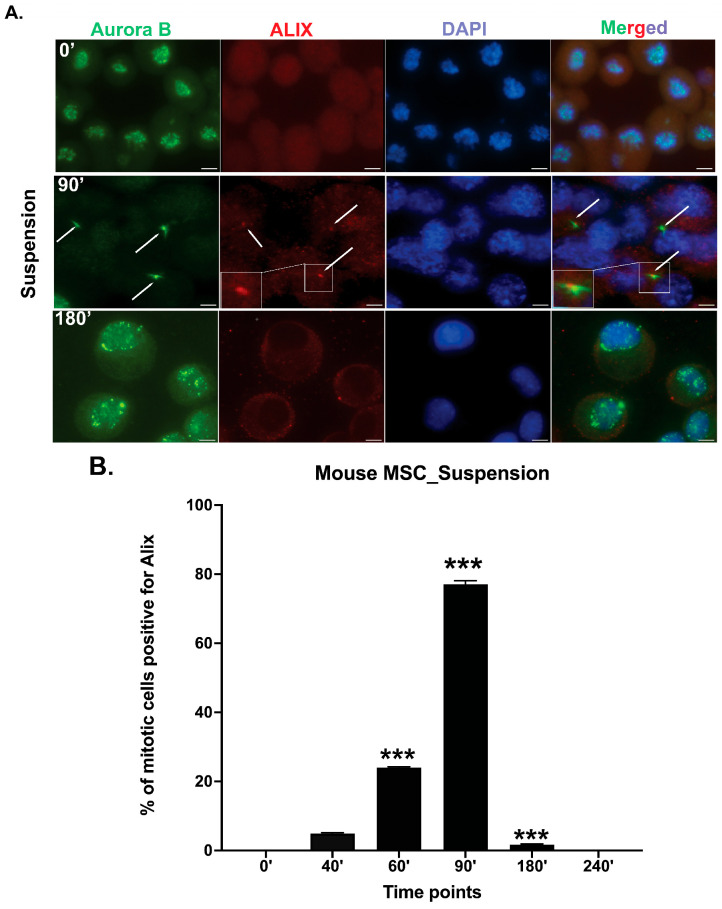
Cytokinesis progresses efficiently in mbm-MSCs under suspension conditions. (**A**) Representative immunofluorescence images of mbm-MSCs, showing the localization of the Aurora B kinase (green), an early midbody marker, and ALIX (red), an abscission-licensing factor, at indicated time points under suspension conditions. Nuclei were counterstained with DAPI (blue). White arrows indicate the midbody region during cytokinesis. The boxed area highlights a magnified view of the midbody region indicated by the arrows. Scale bar: 5 μm. (**B**) The quantification of the percentage of cells displaying the ALIX localization at the midbody over time under suspension conditions. ALIX was detected at the Aurora B-positive midbody in approximately 77% of cells at 90 min, declined to <2% by 180 min, and was undetectable by 240 min, which is consistent with the progression through abscission and the completion of cytokinesis. Data represent mean ± SEM from n = 3 independent experiments. The statistical significance was assessed using an unpaired two-tailed *t*-test; *p* < 0.05 was considered significant. *** *p* < 0.001.

Interestingly, mbm-MSCs cultured in the suspension exhibited temporal dynamics of the Aurora B and ALIX localization similar to those observed under adherent conditions. ALIX was detectable in a subset of cells by 40 min, and by 90 min, 77% of cells showed an ALIX recruitment to the midbody. The number of cells exhibiting Aurora B and ALIX-positive midbodies gradually diminished, becoming undetectable by 240 min. These kinetics suggest that the midbody formation and abscission occur efficiently even in the absence of integrin-mediated adhesion signaling (Figure 4).

### 3.3. Abscission Completes in Suspension-Cultured Mbm-MSCs as Revealed by Re-Adhesion Assay

To confirm that mbm-MSCs in the suspension completed cytokinesis rather than remaining as two cell bodies connected by unresolved intercellular bridges, a replating assay was performed on fibronectin-coated dishes, as previously described [27]. Cells were synchronized at the prometaphase using nocodazole and maintained in the suspension (Pluronic-coated dishes) for 4 h to ensure that midbody structures had been removed. After this incubation, cells were replated onto fibronectin-coated surfaces and monitored by live-cell microscopy to assess the physical separation of daughter cells upon reattachment.

**Figure 5:**
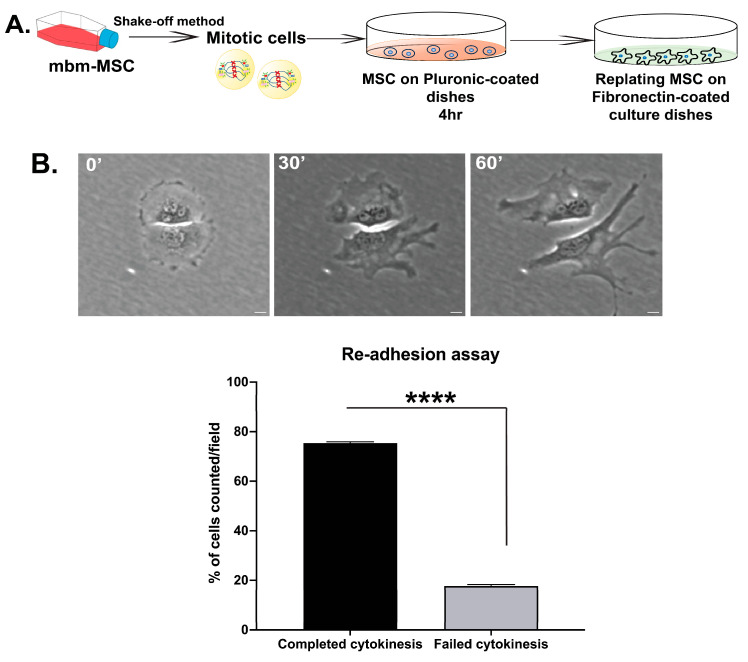
The re-adhesion of mbm-MSCs after cytokinesis in the suspension. (**A**) *Mouse* MSCs were arrested in mitosis with nocodazole and isolated by the shake-off. Mitotic cells were held in suspension for 4 h on Pluronic-coated, non-adhesive dishes, then replated onto fibronectin-coated substrates and monitored by time-lapse microscopy. (**B**) Bright-field images of a representative single MSC at different time points after re-attachment. Mitotic mbm-MSCs were cultured in suspension for 4 h and replated immediately on fibronectin-coated dishes. After the re-attachment, the daughter cell separated without a midbody and there was an observable cytokinetic bridge, demonstrating that the division was completed during the suspension period. The accompanying bar graph quantifies the percentage of cells that completed cytokinesis while in suspension (mean ± SEM, n = 3 independent experiments; ≥100 cells per experiment). Scale bar: 10 μm. The statistical significance was assessed by a paired two-tailed Student’s *t*-test; **** *p* < 0.00001. Scale bar: 10 μm.

Following adhesion, the majority of cells (75.3%) rapidly migrated apart within 30–60 min, indicating that abscission had occurred during the suspension phase before reattachment (Figure 5, Video S3). In some cases, single cells were observed to spread upon replating; and these cells were found to be binucleated, which is consistent with failed cytokinesis and abscission. These findings demonstrate that mbm-MSCs can go through cytokinesis and execute abscission in the absence of integrin-mediated adhesion.

### 3.4. Human Bone Marrow MSCs Complete Cytokinesis in Suspension Independently of Adhesion

**Figure 6:**
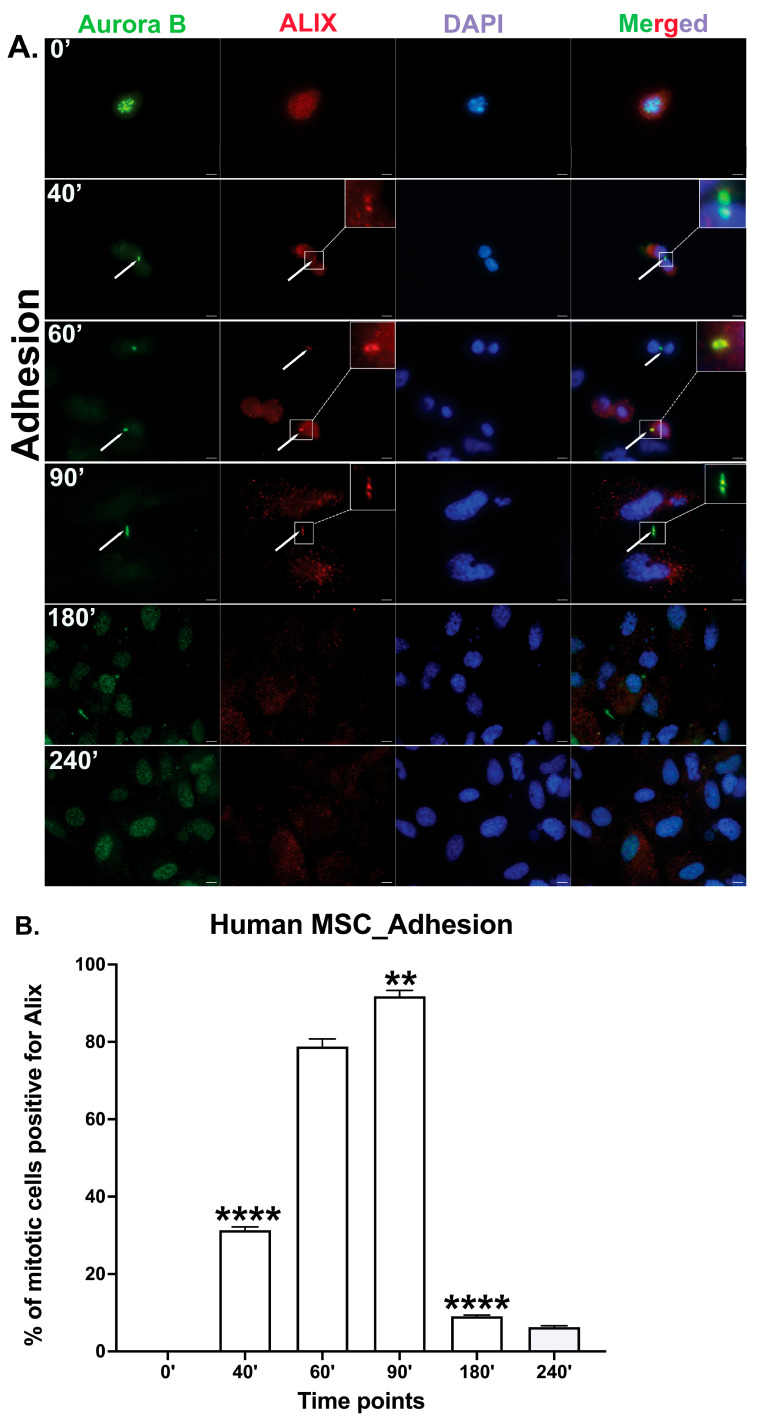
Cytokinesis progresses efficiently in hbm-MSCs under adherent conditions. (**A**) Representative immunofluorescence images of human bone marrow-derived MSCs (hbm-MSCs), showing the localization of Aurora B kinase (green), an early midbody marker, and ALIX (red), an abscission-licensing factor, at indicated time points under adherent conditions. Nuclei were counterstained with DAPI (blue). White arrows indicate the midbody region during cytokinesis. The boxed area highlights a magnified view of the midbody region indicated by the arrows. Scale bar: 5 μm. (**B**) The quantification of the percentage of cells displaying ALIX localization at the midbody over time. ALIX was detected at the Aurora B-positive midbody in approximately 92% of cells at 90 min, declined to <10% by 180 min, and was undetectable by 240 min, indicating the cell cycle progression through abscission and the completion of cytokinesis. Data represent mean ± SEM from n = 3 independent experiments. The statistical significance was assessed using an unpaired two-tailed *t*-test; *p* < 0.05 was considered significant. ** *p* < 0.01 and **** *p* < 0.00001.

**Figure 7:**
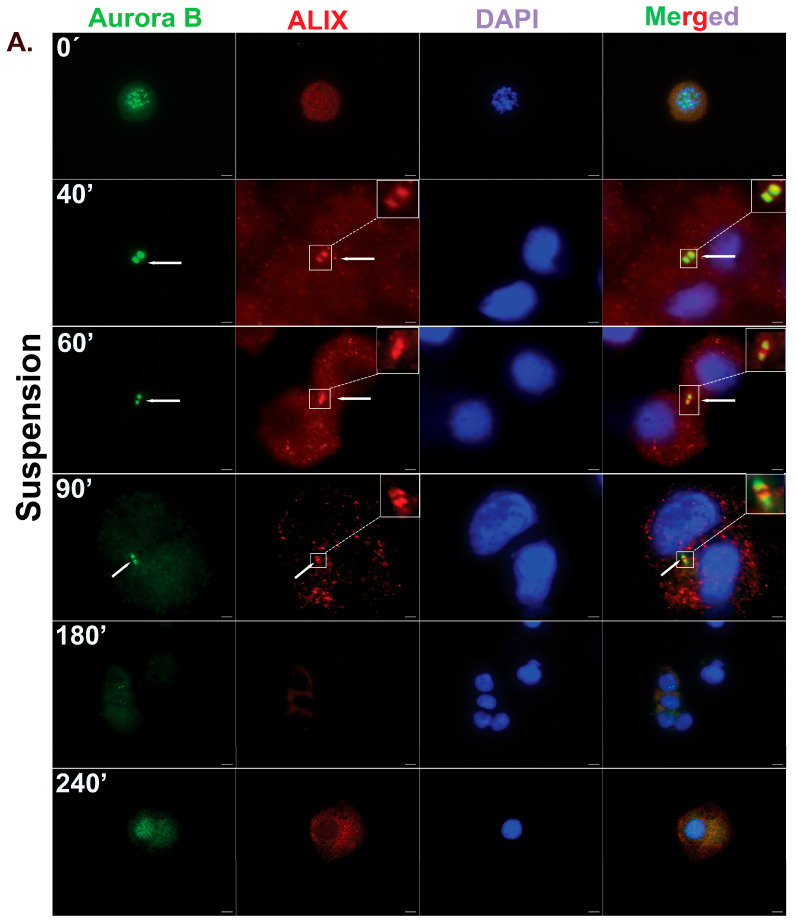

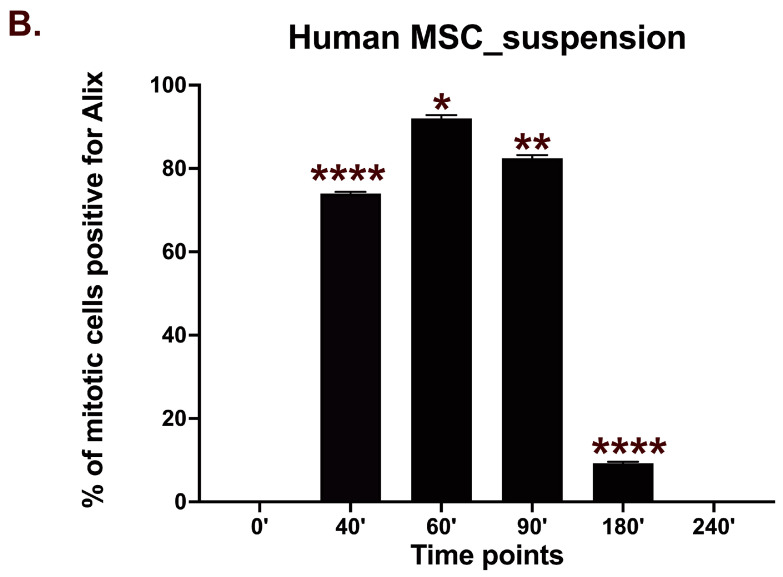
Cytokinesis progresses efficiently in hbm-MSCs under suspension conditions. (**A**) Representative immunofluorescence images of primary *mouse* bone marrow-derived MSCs (mbm-MSCs), showing the localization of Aurora B kinase (green), an early midbody marker, and ALIX (red), an abscission-licensing factor, at indicated time points under adherent conditions. Nuclei were stained with DAPI (blue). White arrows indicate the midbody region during cytokinesis. The boxed area highlights a magnified view of the midbody region indicated by the arrows. Scale bar: 5 μm. (**B**) The quantification of the percentage of cells displaying ALIX localization at the midbody over time under suspension conditions. ALIX was localized to the Aurora B-positive midbody in approximately 92% of cells at 60 in and 82.5% at 90 min. Its presence declined to below 10% by 180 min and became undetectable by 240 min, which is consistent with the successful abscission and completion of cytokinesis. Data represent mean ± SEM from n = 3 independent experiments. The statistical significance was determined using an unpaired two-tailed *t*-test; * *p* < 0.05, ** *p* < 0.01, and **** *p* < 0.00001.

To determine whether *human* bone marrow-derived mesenchymal stem cells (hbm-MSCs) possess a similar capacity to complete cytokinesis under non-adherent conditions, we adopted the same experimental approach as for *mouse* MSCs. hbm-MSCs were synchronized at the prometaphase using nocodazole and then cultured either on fibronectin-coated dishes (adhesive condition) or on Pluronic-coated non-adhesive bacterial-grade dishes (suspension condition). Cells were fixed at specific time points and immune-stained for Aurora B (early midbody marker) and ALIX (key licensing abscission).

hbm-MSCs, like *mouse* MSCs, exhibited the translocation of Aurora B from chromatin (at the 0 min time point) to the midbody, followed by the ALIX recruitment, as the cells progressed through cytokinesis. Under adherent conditions, hbm-MSCs showed the recruitment of ALIX to the midbody in approximately 31.35% of cells by 40 min. This was followed by a peak of ALIX recruitment (approximately 92%) at 90 min, and the subsequent completion of cytokinesis, as both Aurora B- and ALIX-positive midbodies had disappeared by 240 min. (Figure 6). In suspension conditions, the ALIX recruitment to the midbody also occurred, with a peak at 60 (92%) and 90 (82%) minutes, followed by the disappearance of the midbody structure at 180 and 240 min (Figure 7). These results parallel those observed in primary *mouse* MSCs and confirm that hbm-MSCs can complete cytokinesis efficiently without the need for integrin-mediated adhesion. Notably, the kinetics of the ALIX recruitment to the midbody were moderately accelerated in the non-adherent conditions.

### 3.5. Re-Adhesion Assay Confirms Abscission Completion in Suspension-Cultured Hbm-MSCs

To confirm that hbm-MSCs complete abscission during the suspension culture period, we performed the replating assay (described above). Cells were cultured for 4 h in the suspension, to allow sufficient time for the midbody resolution. Upon the reattachment to fibronectin-coated dishes, approximately 61% of the cells exhibited a rapid separation into two distinct daughter cells within 30–60 min of re-adhesion, without the presence of a cytokinetic bridge, indicating that abscission had occurred during the suspension.

**Figure 8:**
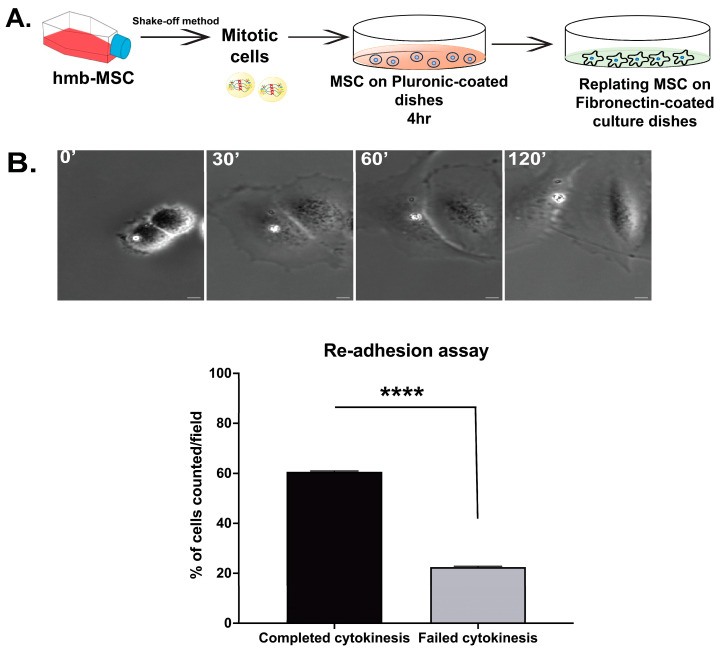
The re-adhesion of hbm-MSCs after cytokinesis in the suspension. (**A**) Human MSCs were arrested in mitosis with nocodazole and isolated by the shake-off. Mitotic cells were cultured in suspension for 4 h on Pluronic-coated, non-adhesive dishes, then replated onto fibronectin-coated substrates and monitored by time-lapse microscopy. (**B**) Bright-field images of MSCs after re-attachment, showing the daughter cell separation without a midbody and an observable cytokinetic bridge, demonstrating that cytokinesis was completed during the suspension period. The bar graph quantifies the percentage of cells that completed cytokinesis while in suspension (mean ± SEM, n = 3 independent experiments; ≥100 cells per experiment). Scale bar: 10 μm. The statistical significance was assessed by a paired two-tailed Student’s *t*-test; **** *p* < 0.00001.

A minor population of cells remained unseparated and appeared as binucleated upon reattachment as a result of incomplete cytokinesis. These results support the conclusion that hbm-MSCs, like the mbm-MSC, are capable of completing cytokinesis and abscission in the absence of integrin-mediated adhesion or mechanical signals from the extracellular matrix. (Figure 8, Video S4).

## 4. Discussion

The single-cell-derived sphere formation is commonly used as an assay for stem cell phenotypes. Consistent with this view, the ability of single cancer cells to proliferate anchorage independently was shown to correlate with a high aldehyde dehydrogenase activity, which is considered to be a stem cell marker [28]. However, this is not a general stemness property since MSCs cannot proliferate in suspension [17]. MSCs share this inability and many other features with fibroblasts, and these cell types have been found to be indistinguishable based on the morphology and the classical marker proteins [29]. Still, more recent studies revealed important molecular differences between primary *human* vascular wall-derived MSCs and primary dermal fibroblasts [30]. By global gene expression and DNA methylation analyses, more than 1000 differentially expressed genes were identified, and notably, a major difference between the cell types was a strong signature for KRAS signaling in MSCs [30]. RAS has been shown to be important for stemness maintenance, and activating RAS mutations induces the formation of cancer cells with stem cell properties [31]. In this context, it is interesting that *human* BJ fibroblasts expressing the mutant HRAS, in contrast to normal BJ fibroblasts, can complete cytokinesis under non-adherent conditions [27]. While the detailed mechanisms for adhesion-independent cytokinesis remain to be identified as well as their possible connection to RAS activity, it is clear that the adhesion-independent cytokinesis ability is a trait that distinguishes MSCs from fibroblasts and thus may be a more general stem cell trait than the spheroid formation ability.

An interesting question then arises: why can several stem cells, in contrast to other cells, complete cytokinesis independently of integrin signals? One hypothesis close at hand is that it provides these cells with a readiness to re-establish their functions after their mobilization to a new location. Regarding bone marrow MSCs, their ability to enter the blood circulation and home to damaged tissue is believed to promote regeneration by several mechanisms, including MSCs’ proliferation and differentiation into organ-specific cell types, the paracrine secretion of bioactive molecules, and the dampening of inflammation [18]. The great potential for clinical applications to mimic the endogenous process by autologous or allogenic transplantation of MSCs has been limited by the low yield of cells engrafting at the desired tissue after systemic administration. Strategies to improve the efficiency have largely been focused on the different steps in the homing process, such as the adhesion to and transmigration through the endothelium at the injured tissue, and guided chemotaxis to the target site [18]. It has also been shown that the expansion of *human* MSCs on a rigid substrate (e.g., plastic dishes) before the transplantation leads to a decline in stem cell properties and therapeutic efficiency, while the suspension culture of cells aggregated into spheres better maintains these features [32,33]. This problem may be related to the high mechanical stress on the cells and the loss of cell fragments when grown on stiff surfaces, as seen in our Video S2. A compliant surface (0.5 kPa) did not induce the tensed morphology (Video S1) but still promoted the cell proliferation to a similar rate or even a somewhat higher rate, and therefore, it may be an alternative to the suspension culture of spheroid aggregates, which has the limitation of cell necrosis in the spheroid center. Another possible obstacle may be the death of the injected MSCs during their non-adherent time while in circulation and before the formation of sufficient integrin contacts at the new tissue environment, like the known low survival rate of tumor cells in the circulation during the metastasis process. One way to improve the transplantation efficiency of MSCs could be to promote a rapid establishment at the foreign targeted site by enabling early ECM deposition and thereby the generation of survival signals at the nascent niche. This could potentially be achieved by the enrichment of cells in the S/G2 phase of the cell cycle by a short growth factor starvation period prior to the harvesting and subsequent administering of the cells. With such a strategy, the cells would proceed directly to mitosis and cytokinesis at the target site even before proper integrin contacts and signaling have been established (i.e., not halted at the G1/S checkpoint), and the two daughter cells would together initiate the assembly of an extracellular matrix more efficiently than a single cell can. In particular, fibronectin polymerization occurs primarily between neighboring cells since it requires the exposure of cryptic FN–FN binding sites by pulling the force from two directions on the dimeric FN protein by myosin via the actin–talin–integrin α5β1 linkage [34,35,36], and it is, therefore, inefficient (or absent) on single non-adherent cells. Thereby, survival and proliferation would be promoted by cytokinetic abscission.

## 5. Conclusions

(1) The capacity to perform cytokinesis independently of adhesion is a trait that distinguishes MSCs from fibroblasts.
(2) While the ability to form single-cell-derived spheres is a functional characteristic of some stem cells including cancer stem cells, but not for MSCs, the capacity for adhesion-independent cytokinesis may offer a more general trait of stemness.
(3) MSCs proliferate well on compliant surfaces and do not exhibit the stress seen on rigid surfaces.

Future research should aim to elucidate the underlying mechanisms of the cytokinesis process in MSCs and other stem cells and explore its implications for enhancing MSC transplantation strategies. In addition, the observed efficient proliferation of MSCs on compliant FN surface motivates investigations regarding their maintenance of functions required for regenerative and immune-suppressive therapies.

## Data Availability

The data presented in this study are available on request from the corresponding author.

## Supplementary Materials

The following supporting information can be downloaded at https://www.mdpi.com/article/10.3390/cells14120932/s1, Video S1: Time-lapse movie of mbm-MSC on soft matrix; Video S2: Time-lapse movie of mbm-MSCs on a stiff substrate; Video S3: Time-lapse movie of mbm-MSC re-adhesion assay; and Video S4: Time-lapse movie of hbm-MSC re-adhesion assay.

## Abbreviations

The following abbreviations are used in this manuscript:

MSC	Mesenchymal stem cell
ALIX	Apoptosis-linked gene 2-interacting protein
mbm	*Mouse* bone marrow
hbm	*Human* bone marrow
