# Supplementary material for: Cytokinesis in Suspension: A Distinctive Trait of Mesenchymal Stem Cells
Source: Cells. 2025 Jun 19;14(12):932. doi: 10.3390/cells14120932 (PMC12191188; doi:10.3390/cells14120932)
Supplement: Supplementary file 1 [file cells-14-00932-s001.zip › Supplementary Data_MSC manuscript 250522.pdf]

Article

Cytokinesis in Suspension: A Distinctive Trait of Mesenchymal Stem Cells

Supplementary Data

Video S1: Time-lapse movie of mbm-MSC on soft matrix demonstrates active cell proliferation on the soft matrix. Scale bar: 10 μm

Video S2: Time-lapse movie of mbm-MSCs on a stiff substrate demonstrates active cell proliferation in adherent culture. Notably, cells exhibited membrane fragments or "footprints" during migration, likely due to increased cytoskeletal tension on the rigid surface.

Video S3: Time-lapse movie of mbm-MSC, showing the daughter cells migrating apart after completing cytokinesis in suspension culture. Scale bar: 10 μm

Video S4: Time-lapse movie of hbm-MSC, showing the daughter cells migrating apart after completing cytokinesis in suspension. Scale bar: 10 μm.
